# Awareness of Family Caregiver Resources and Alignment with Traditional Culture in a Midwest Tribal Nation in the United States

**DOI:** 10.1007/s10900-026-01589-4

**Published:** 2026-06-16

**Authors:** Mary F. Wyman, Danielle B. Lennon, Jesse Montoure, Lauren W.Y. McLester-Davis, Colin Hammock, Nickolas Lambrou, Sacheen Lawrence, Debra A. Miller, Florence Petri, Megan Zuelsdorff, Carey E. Gleason

**Affiliations:** 1 School of Medicine and Public Health, University of Wisconsin – Madison, Madison, WI, USA; 2 William S. Middleton Memorial Veterans Hospital, US Department of Veterans Affairs, Madison, WI, USA; 3 Wisconsin Alzheimer’s Disease Research Center, University of Wisconsin – Madison, Madison, WI, USA; 4 Native American Center for Health Professions, University of Wisconsin – Madison, Madison, WI, USA; 5 Department of Biochemistry, University of Wisconsin – Madison, Madison, WI, USA; 6 Feinberg School of Medicine, Northwestern University, Chicago, IL, USA; 7 Oneida Nation Alzheimer’s Disease Community Advisory Board, Oneida, WI, USA; 8 University of Wisconsin – Madison School of Nursing, Madison, WI, USA

**Keywords:** American Indian(s)/Alaska Native(s), Native or indigenous populations, Family caregiving, Service utilization, Cultural values, Older adults, Alzheimer’s disease and related dementias, Public health service awareness

## Abstract

Family caregivers helping adults with functional needs, including older adults, can benefit from training, support groups, and other services. However, these resources are often underutilized. Caregiving is particularly common in Indigenous communities, but little is known about how Indigenous caregivers consider utilization of supportive resources. To guide efforts to increase utilization, this study sought to describe awareness and attitudes regarding caregiver services within a Midwest Tribal Nation in the United States. Working within a tribally-led, community-based participatory research framework, an online survey was offered to all Tribal members and affiliates aged ≥ 18 years, covering caregiver status, resource awareness and source of information, and beliefs about service use alignment with traditional cultural values. We examined associations between selected factors and awareness. Participants (N = 481) had a mean age of 52.78 (16.6) years, were primarily female (75.5%), and resided both on and off reservation. Almost half (n = 203; 43%) were current caregivers, of whom the majority helped an older adult (n = 173; 83.6%) and/or someone with memory loss (n = 127; 61.4%). Current caregivers, older adults, and reservation residents reported the highest levels of awareness. A significant proportion of respondents (17%) indicated no awareness of any of the 7 resources queried. Primary sources of information for caregivers included independent research and social networks; for non-caregivers, Tribal communications were cited most frequently. Perceptions of alignment of resource use with traditional culture varied by service. Findings add to our understanding of Indigenous family caregiving and can inform public outreach and interventions to increase use of caregiver services.

## Introduction

An estimated 53 million people in the United States (US) currently serve as family caregivers, providing assistance on the basis of their relationship with the care recipient – rather than through professional employment – and typically unpaid for their efforts [1]. Family caregivers represent an invaluable component of the care system for older adults. The experiences, benefits and burdens associated with caregiving have been examined in a rich research literature developed over several decades (e.g., [2]), especially for those helping older adults, who comprise the largest group of caregivers. Resources such as support groups, counseling, respite services, and caregiver training have been shown to reduce caregiver stress and extend the ability to age in place at home [3–5]. In addition, instrumental assistance such as transportation, financial support, and timely access to residential care can offset economic burdens of caregiving [6] and improve care recipient outcomes [7]. Although these resources are generally endorsed as desirable by family caregivers, only a small percentage of caregivers use them [1, 8].

There is a growing impetus to address the needs of those caring for older adults in Indigenous communities. Overall, this population is aging more rapidly than other ethnoracialized groups, such as non-Hispanic Whites [9], and Indigenous Elders are disproportionately impacted by disabling chronic diseases which necessitate daily assistance [10, 11], including dementia [12, 13]. Research suggests that family caregiving is common in North American Indigenous communities [14–16] and is associated with positive benefits, such as strengthening intergenerational relationships [17], but also with high rates of caregiver stress and burnout [18]. Although increasing resource utilization in historically underserved groups has been identified as a critical public health strategy [19], there has been limited research on factors that may impact utilization by Indigenous caregivers [20, 21]. Gaining a better understanding of these factors is critical in order to guide the development of effective interventions to increase utilization and improve outcomes.

Recent work has demonstrated that awareness of services is a key factor in caregiver resource utilization [22]. Among Indigenous Elders, lack of knowledge has been identified as a barrier to healthcare service use [23]. Factors influencing awareness of services may include caregiver residence (e.g., rural vs. urban [24]), gender identity [25], and education level [26]. Furthermore, cultural beliefs and practices can influence decision-making regarding healthcare utilization in Indigenous communities [27, 28]. Important efforts have been made to tailor interventions for Indigenous caregivers [29]; however, there has been limited attention to resource awareness and culture-specific aspects that may affect decisions regarding use.

The current study sought to address these gaps through a research collaboration with one Tribal Nation based in the Midwestern US, informed by community-engaged participatory research (CBPR) and Tribally-led approaches [30]. The study had three aims: (1) describe awareness and use of caregiving-related resources, including sources of information; (2) test associations between demographic characteristics and resource awareness; and (3) explore perspectives on the alignment of traditional cultural values and practices with resource utilization. The primary goal of the study was exploratory and hypothesis-generating. Based on past work in this area, however, we expected that current caregivers would report higher levels of awareness of services, and that perspectives on the alignment of traditional culture with resource use would vary by the type of service.

## Method

### Tribal and Institutional Approvals

Indigenous people have endured a sordid history of unethical medical practices e.g., forced hysterectomies; [31] and unethical research e.g., starvation of children in residential schools; [32] which have been inflicted on them by governmental policies [33, 34]. Due to this history, there is a mistrust among Indigenous people and Tribal governments regarding research participation [33]. Academic institutional review boards attempt to protect individual participants, but often fall short in protecting the interests of Tribes and Tribal data and knowledge [35]. The current study was conducted in partnership with the Oneida Nation, a sovereign Tribal Nation originally based in the eastern US but forced to relocate to the Midwest in 1822 due to colonization of their ancestral lands. The Oneida Nation is now the most populous Tribe in the state of Wisconsin with over 17,000 enrolled members (Oneida Nation Enrollment statistics, 2025). We worked within a CBPR framework, which seeks to establish long-term partnerships and prioritizes community knowledge and engagement throughout the research process [36]. CBPR can help level power dynamics between researchers and community and improve the quality of the research. Adaptations to CBPR strategies for a Tribal context were undertaken in ongoing dialog, including acknowledging Tribal sovereignty and addressing issues of data control [37]. An important structure within this framework is the Oneida Alzheimer’s Community Advisory Board (CAB), formed five years prior to this study and comprising community members, thought leaders, health and aging services leadership, military veterans, and Elders. The lead author (MW) sought CAB input on a proposal to adapt and test a novel caregiver support intervention in the Oneida community. CAB representatives shared their observations that caregiver services already available in the community were underutilized, even when many family caregivers were experiencing obvious struggles and hardships. These services include caregiver support groups, respite, and an evidence-based, culturally-tailored caregiver education program [29], Tribe-supported transportation and household assistance for Elders on or near the reservation, and a senior housing complex with a nursing home located centrally on the reservation. CAB members expressed the desire to understand why caregiver resources went unused and what could help address this problem. Moreover, they emphasized the importance of hearing the voices of Oneida caregivers who live outside the reservation boundaries and/or are not enrolled Tribal members (e.g., non-enrolled descendants or spouses). In order to shed light on this issue, funding was sought and obtained for the Oneida Helping Oneida Family Caregiving Study.

Before the start of research activities, the study protocol was reviewed and approved by the CAB and subsequently reviewed and approved by the Oneida Nation Tribal government, according to established processes (Oneida Business Committee Resolution # 10-28-20-C). Additionally, the research was approved by the University of Wisconsin institutional review board. All participants provided informed consent.

### Participants and Setting

The anonymous online survey was conducted in early 2021. All Oneida Nation community members were eligible regardless of where they resided, including (a) enrolled Tribal members, (b) close family of enrolled members (e.g., non-enrolled spouses), and (c) non-enrolled descendants of enrolled members. Recruitment occurred through social media, the Tribal newspaper, posted flyers (on the reservation and at a social services center in a large metropolitan area to which many Oneida had relocated), and word of mouth. A paper version of the survey with postage-paid return envelope was available upon request. Optionally, participants were offered entry into a cash raffle that required participants to provide a name and email or telephone number. Identifying information was immediately separated from survey responses during initial data processing (*n* = 404 raffle entries). Of a total of 507 responses, *n* = 19 were completed on paper. Data cleaning resulted in *N* = 481 participants with usable data.

### Measures

Survey questions were developed in collaboration with members of the Oneida Alzheimer’s CAB and other local partners (e.g., Oneida Aging & Disability Services and the Oneida dementia care specialist) and after review of a recent community needs assessment. Item format was primarily multiple-choice with free text response options included. The survey was administered within the first year of the COVID-19 pandemic, which brought cancellation, restriction, and/or modification of many caregiver resources (e.g., pausing home respite services; moving support group meetings online). Such changes varied substantially across time and across location within the US in response to virus mutations and local or institutional policy decisions [38]. To contextualize survey responses in light of these disruptions, questions about awareness and use included options specific to the COVID-19 pandemic.

#### Demographics and Caregiver Status

Participants were asked about Tribal affiliation status, residence, and demographics including gender identity, age in years, educational attainment, marital status, employment status, and past or current military service. To identify current family caregivers, we provided a definition (“A family caregiver is a relative, partner, friend, or neighbor who provides assistance for an adult with disabling health conditions, including Elders who need help”), then asked all participants “Are you providing any kind of help or assistance to an adult family member or friend?” Those who responded “yes” were considered current caregivers. Those who responded “no” were identified as non-caregivers and were asked if they knew enrolled members or others who were currently caregiving.

#### Awareness of Services and Source of Information

The survey asked about seven specific resources for caregivers and their care recipients: caregiver support groups, training to help a caregiver take care of an adult family member, respite services (“any service that takes care of an adult family member, so that the caregiver can take some time away from helping”), financial help (including helping the care recipient apply for Medicaid or other financial programs), residential or nursing home facilities, transportation services for the care recipient, and household help (e.g., cooking, cleaning, yardwork). The first four services were based on items from the National Study of Caregiving (NSOC), a longitudinal, nationally representative survey of informal caregivers linked to the National Health and Aging Trends Study [39]. Three additional resources were included based on community input. For each resource, participants were asked, “Do you know about the resource and how someone would access it?” Multiple choice response options included: *(1) I know about it and how to access; (2) I know about it, but not how to access during the COVID-19 pandemic; (3) I know about it but never knew how to access; and (4) I don’t know about it and don’t know how to access*. Participants reported knowledge of resources in their own community, whether they lived on the Oneida reservation or elsewhere. For analyses of group differences, we dichotomized responses into *“aware”* (options 1–3) and *“not aware”* (option 4).

For the four resources derived from the NSOC, if participants indicated they were aware of the service, they were asked how they learned about it. Multiple sources could be endorsed. Options included: *talking to a medical care provider or social worker; church or other spiritual community; On your own – e.g., from a friend/family, online, or at the library; local Tribal communications (e.g., newsletter or flyer); your employer;* or *any other source* with optional free-text response. A team of researchers and community members used a consensus approach to review and re-code free-text responses to existing categories as appropriate.

#### Perceptions Regarding Cultural Traditions and Service Use

To gain insight into perceptions of the alignment of service use with this community’s culture, we asked all survey participants: “We are interested in your opinion about how much the traditional values and practices of the Oneida Nation support family caregivers in using resources to help them in the caregiving role. In other words, how consistent is Oneida traditional culture with people accepting services to help them take care of family members or friends who need assistance?” For each of the seven resources, response options were *not at all, a little, somewhat, very much* or *don’t know/prefer not to answer*.

### Data Analysis

We used descriptive statistics to characterize the sample overall and by caregiver/non-caregiver status, including rates of resource awareness and sources of information. Next, using the dichotomized awareness variable, we examined differences in awareness of each resource by participant characteristics chosen based on previous research, including caregiver status, residence location (within vs. outside of reservation boundaries), gender identity (female vs. male/two-spirit/other) [40], education level (≤ high school vs. > high school), and age using Fischer’s exact test or t-test, as appropriate for variable type. Finally, we described perceptions of alignment of use with traditional culture and values for each queried service. Analyses were conducted with IBM SPSS Statistics for Windows, Version 29.0.

## Results

### Participants

Of the 481 survey participants, 432 (89.9%) identified as enrolled members of the Oneida Nation. Average age was 53 years (range 18–87), and 363 (75.5%) of participants identified as female, 115 (23.9%) as male, and 2 (0.4%) as two-spirit or other. The sample was geographically diverse, with about half living inside reservation boundaries or in the adjacent counties. Over 10% of participants lived outside of the state (see Table 1).

Of the 207 (43%) who endorsed being a current family caregiver, 61.8% lived outside of reservation boundaries, and 12.6% were non-Tribal members. Most caregivers were helping an older adult (*n* = 173, 83.6%) and most reported that the care recipient had memory loss (*n* = 127, 61.4%). Among non-caregivers (*N* = 274), 44.6% reported that they personally knew an enrolled Oneida Tribal member who was providing assistance to an Elder, and 11.2% knew a non-enrolled descendant or family member who was providing assistance to an Elder.

### Awareness of Services

**Fig. 1:**
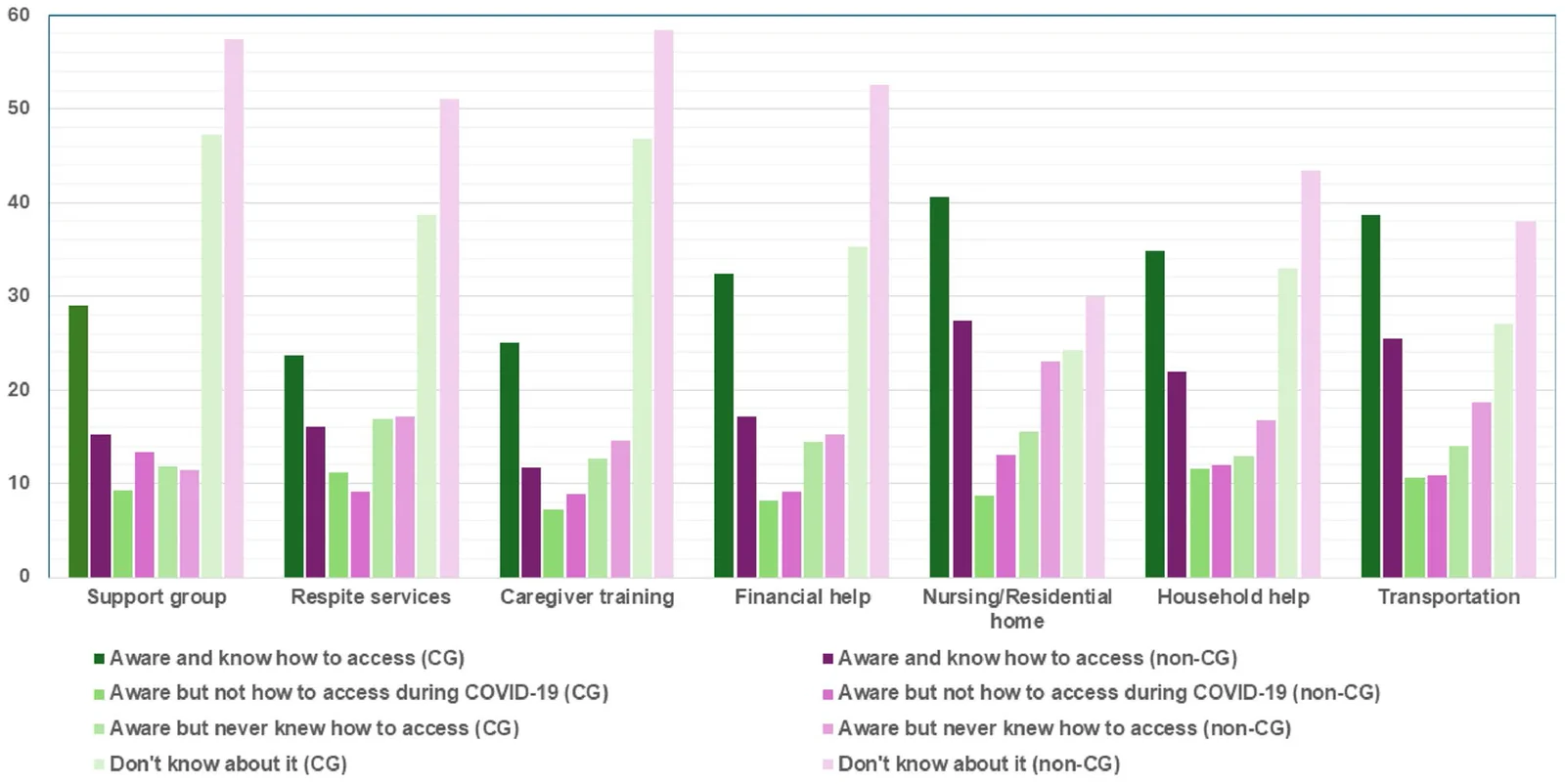
Awareness level of caregiving resources by caregiver or non-caregiver status (%). Percentages shown indicate proportions of caregivers (CG; *N* = 207) or non-caregivers (non-CG; *N* = 274) endorsing each level of awareness. Multiple answers for each item were accepted. Missing values range from 1.1–2.9% for each resource

Figure 1 shows awareness of and how to access each resource, presented by caregiver status. Among caregivers, awareness was highest for the existence of nursing homes or residential facilities, with 40.6% reporting they knew about this resource and how to access it, while respite services were least known to caregivers (23.7% know about it and how to access). Compared to caregivers, non-caregivers showed generally lower levels of awareness, with nursing homes being most known (27.4%) and caregiver training least well known (11.7%). Overall, only a modest percentage indicated they knew about a service but did not know how to access it during the COVID-19 pandemic (range 7.2–13.4%). A larger proportion of the sample endorsed no awareness of resources (range 24.2% – 58.4%), with over a third of caregivers, and more than half of non-caregivers, reporting no awareness of four out of the seven services included in the survey.

### Differences in Awareness

When awareness of services was examined using the dichotomized variable (aware vs. not aware), most participants reported awareness of nursing home services (*n* = 308, 64.0%) and transportation (*n* = 282, 58.6%), while a minority were aware of caregiver training (*n* = 189, 39.3%) and support groups (*n* = 204, 42.4%). There were 81 participants (17%) who were not aware of any of the seven listed services.

Table 2 shows results of group comparisons on awareness. Compared to non-caregivers, more participants who were current caregivers reported awareness of six of seven resources. There was no statistical difference by caregiver status in awareness of nursing/residential home resources. More participants who lived within reservation boundaries reported awareness of nursing/residential home resources, household help, and transportation services compared to those who lived off reservation. There were no differences in awareness by gender identity or dichotomized education level (≤ high school vs. > high school). Participant age was associated with awareness of support groups (M_aware_ = 55.09 years, SD 16.42 vs. M_not aware_ = 50.9, SD 16.50, *t*(443) = ≥2.65, *p*=.008) and household help (M_aware_ = 54.26 years, SD 16.21 vs. M_not aware_ = 50.97, SD 16.84, *t*(447) = ≥2.09, *p*=.038). Only 36.9% of participants aged 18–39, compared to 54.3% of older adults (aged ≥ 60), endorsed awareness of caregiver support groups (*p* < .01). There were no differences by age in the other resources queried.

### Source of Information About Services

**Fig. 2:**
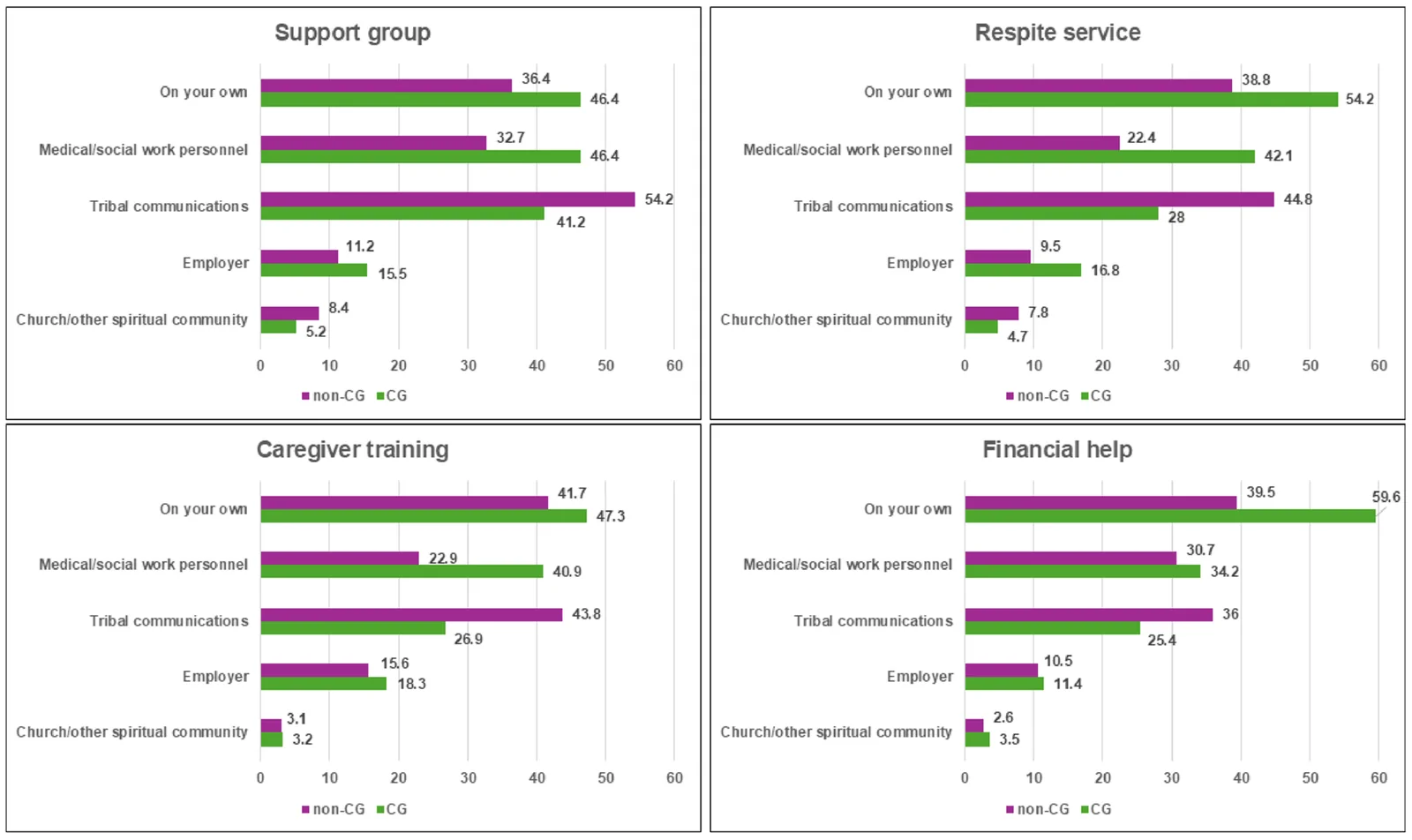
Sources of information about four caregiver resources reported by caregivers and non-caregivers (%). Numbers indicate percentages of caregivers (CG; Ns range from 93–114) or of non-caregivers (non-CG; Ns 96–116) who reported each source. For these four resources derived from the National Study of Caregiving (Freedman et al., 2019), if participants indicated they were aware of the service, they were asked how they learned about it. *On your own* includes “from a friend/family, online, or at the library”; *Tribal communications* includes local newsletters or flyers. Multiple sources could be endorsed. Responses listed as “Any other source” with additional free-text information were re-coded to other categories as appropriate

Figure 2 shows sources of information by caregiver status. Across the four queried services, the most common sources of information were *on your own, medical/social work personnel*, and *Tribal communications*. For caregivers, *on your own* was the top reported source, cited most frequently for three of the resources (respite services, caregiver training, and financial help) and the same as the next most common source, *medical/social work personnel*, for one resource (support group). For non-caregivers, *Tribal communications* was the most frequently reported source of information for three of four resources (support group, respite services, and caregiver training). *Employer* and *church/other spiritual community* were the least cited sources. Review of the text responses revealed diverse pathways for some of these sources. For example, some participants had gained knowledge through working in the Elder care field or had talked to family with relevant work experience. Some noted they had learned about services through their own previous caregiving experience or from talking to friends and neighbors who have been caregivers.

### Perceptions of Alignment of Service Use with Traditional Culture

Ratings by participants on whether traditional culture supports service use varied across service type. For six of the seven resources, roughly half of participants agreed that resource use was *somewhat* or *very much* consistent with traditional cultural values and practices. In general, more caregivers than non-caregivers rated the use of a service as *not at all* consistent with traditional culture, and fewer caregivers than non-caregivers rated service use as *very much* consistent with traditional culture (Table 3). For example, use of nursing home or residential home services was rated *not at all* consistent with traditional culture by 25.3% of current caregivers but only 10.6% of non-caregivers. Notably, compared to other survey questions, these items had a higher rate of *Don’t know or prefer not to answer* responses (on average, 22.6% for caregivers and 23.7% for non-caregivers).

## Discussion

We report on results from a community-wide survey conducted in the Oneida Nation, a Tribal Nation in the Midwestern US, and describe the level of awareness of family caregiver services and perceived alignment of service use with traditional cultural values. Our findings add to the limited literature on family caregiving in Indigenous communities and provide a critical basis for advocacy, policy development, and future research.

Consistent with previous work conducted with minoritized groups [22, 41], we identified low levels of awareness about key resources to support caregivers and care recipients. This aligns with research conducted in other North American Indigenous communities [14] and underlines the need for continued investigations in this area to inform policy that can support outreach. Our finding that non-caregivers reported less awareness than current caregivers was not surprising, given that information pathways typically flow through places where caregivers are already connected. Given that a sizable proportion of non-caregivers knew someone who was caregiving, however, our findings provide support for the use of a broader public health strategy aimed at the community as a whole. Similarly, we found that Oneida Nation members who live off the reservation reported lower levels of awareness than those on the reservation. This points to the need for innovative strategies to reach Tribal members who are not present in local caregiver circles, but who nevertheless are part of the support system for Elders and others receiving care. Our results suggest that this also applies to younger persons, who are increasingly visible in the national family caregiving workforce [42].

Among both caregivers and non-caregivers, service awareness was highest for nursing or residential homes, but lowest for services which are typically mainstays of caregiver interventions to support non-institutionalized care recipients, such as support groups, caregiver training, and respite care [43]. This is notable in light of the fact that most adults who need caregiving assistance live at home [8], and that the growing population of Indigenous Elders places high value on aging in place within their communities [44]. Awareness was higher for services offered by the Tribe, such as transportation and household help, which support Elders at home but are not aimed specifically toward caregivers. This may indicate an opportunity to promote availability of caregiver-specific resources along with such broader services in order to increase awareness and potentially utilization. We did not see evidence that levels of awareness or knowing how to access services were significantly impacted by the arrival of COVID-19. While this event altered the experience of caring for a loved one [45], our results point to barriers to awareness for many in this community that pre-date pandemic restrictions. Previous work has shown that low awareness is a prevalent barrier to utilization of services by caregivers of older adults [22]. Increasing awareness of caregiver resources remains an important target for intervention.

Our findings regarding sources of information about services point to opportunities for medical and social work professionals, employers, and church or spiritual groups to more effectively serve as information hubs for Indigenous families. Our results also highlight the value of online and in-person social networks for promoting resource use. A large literature emphasizes the importance of social relationships for caregiver well-being, and recent work showed a positive association between social networks and service use among caregivers of children with disabilities [46]. However, the potential of social connections to promote service awareness among caregivers of adults and older adults has received limited attention. Future research should further examine how social networks may facilitate Indigenous caregivers learning about services and deciding to use them.

Our findings on participants’ perceptions of how resource use aligns with Oneida traditional cultural values suggest that there may be important differences in how the community’s current caregivers, compared to non-caregivers, view service use in the context of traditional ways. Moreover, we found that ratings of alignment varied by service, consistent with what others have reported [14], in that participants rated use of nursing or residential homes as less aligned than other services. This is notable in light of our finding that awareness was highest for this service, and suggests a mismatch between awareness of this service and preferences for use. Others have noted the importance of culture-based interventions for Indigenous Elders and caregivers to maximize access and effectiveness [27, 47]. For example, “Respect for Elders” is a central cultural value which supports the concept of caregiving within North American Indigenous communities [18]. The authors of the adaptation of the Resources for Enhancing Alzheimer’s Caregivers Health [REACH; 29] caregiver education program for delivery within Tribal Nations reported that while some participants expressed concern that this cultural tradition had eroded over generations, Elder Respect appears to have represented a barrier to engagement for some caregivers, when the Elder’s wishes were the determining factor on whether caregivers were allowed to participate in the REACH program. Our study builds on past work showing that caregiving practices are impacted by culture [16, 48] and points to the need for future examination of the connection between cultural values and decision-making about caregiver resource use.

The Indigenous experience is characterized by resilience, and Indigenous community members bring many strengths to the family caregiver role. Still, there are a myriad of challenges which negatively impact the ability of family to care for Elders, including current circumstances as well as historical trauma [14]. Members of Indigenous communities often must navigate complex care systems in under-resourced settings, sometimes with limited health literacy [49]. In light of the elevated rates of disabling conditions among North American Indigenous Elders [10, 11], caregivers may be more likely to be performing challenging caregiving tasks. Dementia is a major public health issue for Indigenous communities [13], and most of the current caregivers in this study were helping an Elder with memory loss. Among Alaska Natives, a recent study found that lack of understanding and awareness of services, including culturally appropriate resources, negatively impacts the dementia caregiving experience [50]. However, increasing awareness of symptoms of cognitive decline or early dementia and the impact of cognitive decline/dementia on the community may help promote the implementation of caregiver programs [29]. Our findings support the consensus recommendations of a recent report, which highlighted the complex systems that can impact persons with dementia and their families, including resource use [51]. Our results also provide empirical support for policy goals in the National Strategy to Support Family Caregivers [19]. Outreach should incorporate a multi-prong, multi-pathway strategy for information dissemination to increase appropriate use of available resources, and efforts to increase awareness and shift attitudes can target societal, Tribal, and individual levels of identity. Public outreach campaigns could emphasize the ways in which utilization of resources to support caregivers aligns with identity and culture. Collaborations with local Indigenous culture and language revitalization initiatives could positively shift perceptions of service use. Importantly, the benefits of family caring for family should be highlighted. For example, Jacklin and colleagues [14] note that engaging in care of Indigenous Elders facilitates transmission of critical knowledge and values to younger generations, sustaining and preserving culture for the entire community. This can help reduce burden and strengthen personal, familial, and community health [52].

### Limitations

It is critical to pursue location-specific, well-characterized research on Indigenous caregiving, and our findings from a Midwestern Tribal Nation make an important and novel contribution. However, although the sample is demographically diverse, it is not statistically representative of the Oneida Nation and cannot be considered to represent Indigenous communities broadly. Additionally, because the availability of caregiving resources will vary by locality – most services are offered by local, county or state-level programs – we could not differentiate between low awareness of existing resources versus a lack of awareness of a resource because the service doesn’t exist or is not accessible where the participant lives. Within North America and across the globe, there is significant variation across Indigenous communities in geographic characteristics (e.g., distance from service providers), available resources, and cultural approaches to Elder care [53]. This study suggests a number of potentially important influences on service awareness in Oneida, which may represent worthwhile directions for future work with other Tribal Nations. Finally, a quantitative survey methodology has its own limitations. Although we worked closely with our community partners to construct items, a subset of participants declined to answer the questions about alignment of service use with culture. This may indicate low willingness to disclose on a sensitive subject, or may indicate challenges for some participants in answering questions about how service use decisions relate to “traditional culture.” More research is needed, and a qualitative approach would be well-suited to expand our understanding of this topic.

## Conclusion

Family caregiving is common in Indigenous communities. Despite low utilization of services to support caregiver and care recipient, limited attention has been given to factors impacting awareness of such resources. Findings from the current study can help guide efforts to increase the use of community services and resources by Indigenous family caregivers. Findings from our study, co-designed and conducted within a robust community-led approach in one Tribal Nation, suggest the benefits of a broad, public health-based approach to disseminating information about resources to all members of the community – including those who are not currently caregiving, but who know those who are, or will become caregivers in the future. Acknowledging the critical contributions and ubiquity of caregiving in Indigenous communities, and the need for community support for the friends and family who are providing care, is a first foundational step forward.

## Figures and Tables

**Table 1:** Key demographics of survey participants, by caregiver and non-caregiver status

		Overall sample (*N* = 481)	Current caregiver (*N* = 207)	Current non-caregiver (*N* = 274)
*N*				
Gender Identity, N (%)	Female	363 (75.5)	157 (75.8)	68 (75.2)
	Male	115 (23.9)	47 (22.7)	206 (24.8)
	Two-spirit	2 (0.4)	2 (1.0)	
	Other	1 (0.2)	1 (0.5)	
Age in years, Mean (SD)		52.78 (16.60)	51.81 (15.33)	53.51 (17.48)
Educational Attainment, N (%)	Less than HS	18 (3.7)	10 (4.8)	8 (2.9)
	HS diploma/GED	160 (33.3)	65 (31.4)	95 (34.7)
	Associate/trade school	137 (28.5)	55 (26.6)	82 (29.9)
	Bachelor’s	96 (20.0)	42 (20.3)	54 (19.7)
	Professional degree	67 (13.9)	34 (16.4)	33 (12.0)
	No answer	3 (0.6)	1 (0.5)	2 (0.7)
Marital status, N (%)	Single, never married	113 (23.5)	51 (24.6)	62 (22.6)
	Married or living with partner	234 (48.6)	103 (49.8)	131 (47.8)
	Divorced or separated	90 (18.7)	36 (17.4)	54 (19.7)
	Widowed	39 (8.1)	15 (7.2)	24 (8.8)
	Prefer not to answer	5 (1.0)	2 (1.0)	3 (1.1)
Oneida Affiliation, N (%)	Enrolled Tribal member	432 (89.8)	181 (87.4)	251 (91.6)
	Non-enrolled descendant	19 (4.0)	12 (5.8)	7 (2.6)
	Non-enrolled family/close friend of enrolled member (e.g., spouse)	30 (6.2)	14 (6.8)	16 (5.8)
Residence, N (%)	Within reservation boundaries	153 (31.8)	77 (37.2)	76 (27.7)
	Outside reservation boundaries, in adjacent counties	107 (22.2)	41 (19.8)	66 (24.1)
	In large metro area within WI	105 (21.8)	41 (19.8)	64 (23.4)
	Elsewhere in WI	63 (13.1)	33 (15.9)	30 (10.9)
	Outside of WI	50 (10.4)	13 (6.3)	37 (13.5)
	Prefer not to answer	2 (0.4)	2 (1.0)	1 (0.4)
Employment status, N (%)	Employed	241 (50.1)	112 (54.1)	129 (47.1)
	full-time	192 (39.9)	87 (42.0)	105 (38.3)
	part-time	49 (10.2)	25 (12.1)	24 (8.8)
	Furloughed (full or in part) due to the COVID-19	22 (4.6)	9 (4.3)	13 (4.7)
	pandemic	32 (6.7)	18 (8.7)	14 (5.1)
	Unemployed, seeking employment	182 (37.8)	93 (44.9)	116 (42.3)
	Don’t work or retired	4 (0.8)	2 (1.0)	2 (0.7)
	Prefer not to answer			
Past/current military service, N (%)	Yes	36 (7.5)	19 (9.2)	17 (6.2)
	No	443 (92.1)	187 (90.3)	256 (93.4)
	Prefer not to answer	2 (0.4)	1 (0.5)	1 (0.4)

Data were collected Jan - Mar 2021. The self-report survey was completed online by all participants except *n* = 19 completed the survey on paper, per their request.

**Table 2:** Group comparisons of awareness of seven caregiving resources by key participant characteristics

	CG (*n* = 191)^a^	Non-CG (*n* = 258)		Within reservation (*n* = 141)	Off reservation (*n* = 301)		Male, two-spirit or other (*n* = 111)	Female (*n* = 334)		≤ high school (*n* = 163)	> high school (*n* = 283)	

	% aware		*p*-value	% aware		*p*-value	% aware		*p*-value	% aware		*p*-value
Support group	51.6	41.6	**0.043**	51.8	42.9	0.083	43.2	46.7	0.583	46.9	45.4	0.767
Respite services	57.2	45.3	**0.016**	55.8	47.7	0.124	49.5	50.6	0.913	49.1	51.2	0.692
Caregiver training	48.9	37.5	**0.020**	46.0	40.5	0.300	39.3	43.4	0.508	45.1	40.9	0.425
Financial help	44.2	61.0	**< 0.001**	57.1	48.7	0.103	47.3	52.6	0.382	50.6	51.4	0.921
Nursing home/residential home	72.8	68.0	0.293	79.7	65.2	**0.002**	68.2	70.6	0.633	65.0	72.9	0.084
Household help	64.4	53.9	**0.026**	72.5	51.3	**< 0.001**	55.4	59.3	0.507	54.6	60.4	0.233
Transportation services	70.1	59.2	**0.021**	78.3	56.8	**< 0.001**	60.4	65.0	0.425	59.7	66.1	0.215

Percentages show proportion of that group who report awareness of the service, using dichotomized variable (aware vs. not aware). Comparisons used Fisher’s exact test with 2-sided tests of significance (p-values < 0.05 are bolded).

a Due to missing data, total Ns range from 439 to 449.

**Table 3:** Respondent perspectives on alignment of traditional culture with caregiver resource use

	Not at all		A little		Somewhat		Very much		Don’t know/ Prefernot to answer	
*N (%)*										
*How much do you believe Oneida traditional culture supports the use of…*.	CG	Non-CG	CG	Non-CG	CG	Non-CG	CG	Non-CG	CG	Non-CG
Support group	20 (11.2)	22 (8.6)	24 (13.5)	28 (11.0)	41 (23.0)	54 (21.2)	53 (25.6)	86 (33.7)	40 (19.3)	65 (25.5)
Respite services	21 (11.8)	30 (11.8)	24 (13.5)	22 (8.6)	42 (23.6)	53 (20.8)	46 (25.8)	83 (32.5)	45 (25.3)	67 (26.3)
Caregiver training	21 (11.9)	34 (13.3)	25 (14.1)	21 (8.2)	42 (23.7)	51 (20.0)	46 (26.0)	86 (33.7)	43 (24.3)	63 (24.7)
Financial help	25 (14.0)	29 (11.4)	19 (10.7)	19 (7.5)	46 (25.8)	45 (17.6)	49 (27.5)	97 (38.0)	39 (21.9)	65 (25.5)
Nursing home/residential home	45 (25.3)	27 (10.6)	23 (12.9)	47 (18.4)	37 (20.8)	63 (24.7)	30 (16.9)	59 (23.1)	43 (24.2)	59 (23.1)
Household help	24 (13.5)	20 (7.8)	26 (14.6)	38 (14.9)	38 (21.3)	52 (20.4)	54 (30.3)	91 (35.7)	36 (20.2)	54 (21.2)
Transportation services	26 (14.6)	24 (9.4)	18 (10.1)	22 (8.6)	41 (23.0)	56 (22.0)	52 (29.2)	103 (40.4)	41 (23.0)	50 (19.6)

Percentages indicate proportion of caregivers (CG; *N* = 207) or of non-caregivers (non-CG; *N* = 274).

## Data Availability

All information collected within the boundaries of the Oneida Nation is the property of the Oneida Nation. The data supporting these findings can be made available upon reasonable request to the authors and with the approval and authorization of the Oneida Nation.
